# A TNNT2 variant in a sporadic case of dilated cardiomyopathy: a case report and review

**DOI:** 10.3389/fcvm.2026.1757484

**Published:** 2026-03-26

**Authors:** Xinglin Chen, Zhengjie Huang, Qi Zheng, Lingfeng Zha, Tingting Tang, Xiang Cheng

**Affiliations:** 1 Department of Cardiology, Union Hospital, Tongji Medical College, Huazhong University of Science and Technology, Wuhan, China; 2 Hubei Key Laboratory of Biological Targeted Therapy, Union Hospital, Tongji Medical College, Huazhong University of Science and Technology, Wuhan, China; 3 Hubei Provincial Engineering Research Center of Immunological Diagnosis and Therapy for Cardiovascular Diseases, Union Hospital, Tongji Medical College, Huazhong University of Science and Technology, Wuhan, China

**Keywords:** dilated cardiomyopathy, genetic variant, genotype-phenotype correlation, TNNT2, whole-exome sequencing

## Abstract

**Purpose:**

This study aims to characterize the clinical and genetic features of a sporadic case of dilated cardiomyopathy (DCM) associated with a TNNT2 variant and to review the variant spectrum of the TNNT2 gene in the Chinese DCM population.

**Patients and methods:**

A 41-year-old male proband diagnosed with DCM underwent comprehensive clinical evaluation. Whole-exome sequencing (WES) was performed on the proband to identify potential causative variants. Subsequently, Sanger sequencing was used to specifically validate the candidate TNNT2 variant in the proband and all available family members. Bioinformatic tools were employed to predict the pathogenicity of the identified variant, which was interpreted according to the American College of Medical Genetics and Genomics (ACMG) guidelines. A literature review of TNNT2 variants in Chinese DCM patients was conducted.

**Results:**

Clinical assessment revealed left ventricular dilation and systolic dysfunction in the proband. Genetic analysis identified a heterozygous missense variant in the TNNT2 gene (c.311G > A, p.Arg104His). Bioinformatic predictions consistently supported its deleterious effect and high conservation. The variant was not found in the available family members. Considering the maternal sudden death history, it is likely to be *de novo* or maternally inherited. Based on ACMG guidelines, the variant was classified as “Likely Pathogenic”. The literature review summarized 14 distinct TNNT2 variants from 20 reported Chinese DCM cases.

**Conclusion:**

This study provides a detailed clinical characterization of the TNNT2 c.311G > A (p.Arg104His) variant in a Chinese patient with sporadic DCM, contributing to the understanding of its phenotypic spectrum. The review provides an overview of the TNNT2 variant spectrum in the Chinese DCM population.

## Introduction

Dilated cardiomyopathy (DCM) is defined by the presence of left ventricular (LV) or biventricular dilatation and systolic dysfunction in the absence of abnormal loading conditions (e.g., hypertension, valve disease) or coronary artery disease sufficient to cause global systolic impairment (1). It is a leading cause of heart failure and the most common reason for heart transplantation (2). While various factors such as infection, toxins, and metabolic/endocrine disorders can contribute to DCM, genetic variants play a significant role in its pathogenesis. Clinical screening of first-degree relatives—comprising an electrocardiogram, echocardiography, or other assessments of left ventricular size and function—identifies a familial form of dilated cardiomyopathy (FDC) in at least 20%–35% of individuals initially diagnosed with idiopathic DCM (IDC) (3). FDC is primarily inherited in an autosomal dominant manner (4, 5). Identifying genetic variants helps assess disease progression and prognosis, thereby guiding early therapeutic decisions (6–8).

Currently, over 250 genes spanning more than 10 gene ontologies have been implicated in DCM (9). However, the frequencies of DCM variants in any single gene are low (<1% to 6%–8%), and a genetic cause is identified in only 30%–35% of FDC cases (3, 10). The TNNT2 gene is located on human chromosome 1q32 and consists of 17 exons. It encodes cardiac troponin T (cTnT), a critical component of the cardiac troponin complex, where it plays a key role in regulating heart muscle contraction. A clear association between TNNT2 and dilated cardiomyopathy (DCM) has been established (9, 11). The pathogenic mechanisms of TNNT2 variants involve dysregulated sarcomere-mitochondria crosstalk (12), aberrant sarcomere organization, reduced calcium sensitivity, and diminished cardiomyocyte contractility (13).

The TNNT2 c.311G > A (p.Arg104His) variant is well-established as likely pathogenic for hypertrophic cardiomyopathy and has also been associated with dilated cardiomyopathy. However, detailed clinical characterization of this variant in DCM, particularly in Chinese patients, remains limited. Here, we provide a detailed clinical characterization of this variant in a Chinese patient with sporadic DCM through integrated family analysis, clinical imaging, whole-exome sequencing, Sanger sequencing, and bioinformatic prediction. We also systematically review the variant spectrum of this gene in the Chinese DCM population.

## Material and methods

### Study subject

The proband was a 41-year-old male diagnosed with DCM according to the 2023 European Society of Cardiology (ESC) Guidelines for the management of cardiomyopathies (14). The proband reported that his mother died suddenly at approximately 35 years of age (specific cause unknown). Other family members were healthy with no history of cardiac disease. Written informed consent was obtained from all participants.

### Clinical evaluation

Clinical evaluation included detailed medical history, family history, physical examination, 12-lead electrocardiogram (ECG), two-dimensional and Doppler echocardiography, and cardiac magnetic resonance imaging (CMR). Additional tests included complete blood count, immune fixation electrophoresis, quantitative serum and urine light chains, quantitative serum immunoglobulins, and autoantibody panels. The autoantibody panel included anti-adenine nucleotide translocator antibody (ANT), anti-*β*1-adrenergic receptor antibody (*β*1-AR), anti-myosin heavy chain antibody (MHC), and anti-L-type calcium channel antibody (L-CaC).

### Whole-exome sequencing (WES)

Peripheral blood samples were collected from the proband and four relatives (his daughter, half-brother, maternal uncle, and maternal aunt). Genomic DNA was extracted. Libraries were prepared, and exonic regions and flanking splice sites were captured using the Roche KAPA HyperExome kit (Roche Diagnostics). Sequencing was performed on MGISEQ-2000 or DNBSEQ-T7 platforms. Quality control metrics included a mean sequencing depth of ≥200× in the target regions, with >98.5% of bases covered at >20x. Candidate causative variants were initially screened based on WES data.

### Sequence analysis

Sequencing reads were aligned to the UCSC hg19 reference genome using BWA. Duplicates were removed. Variant calling (SNVs, INDELs) and genotyping were performed using GATK. Copy number variations (CNVs) were detected using ExomeDepth. Identified variants were annotated and filtered against multiple population and disease databases, including the NCBI dbSNP (v147), dbNSFP (v2.9.1), the NHLBI Exome Sequencing Project (ESP6500), the 1000 Genomes Project, and an in-house database of 100 ethnically matched healthy controls, using BGI's proprietary annotation pipeline.

### Sanger sequencing validation

Candidate causative variants were validated by Sanger sequencing. PCR amplification was performed using specific primers (Forward: 5'-TTGATGTAAGCGGTGGCTGT-3’; Reverse: 5'-CCAGTAGGATGGGGAGGGAA-3’). Amplified DNA fragments were sequenced by BGI. Chromatograms were analyzed using SeqMan software to confirm the variant and determine zygosity.

### *In silico* prediction

The variant's frequency was checked in population databases including 1000 Genomes, ESP6500, ExAC, gnomAD, and gnomAD-EAS. Pathogenicity was predicted using SIFT, MutationTaster, Condel, and REVEL. Potential splice-altering effects were assessed using SpliceAI, dbscSNV_RF, and dbscSNV_ADA. Evolutionary conservation was analyzed using PhyloP and GERP++ via multiple sequence alignments performed with ClustalX. The three-dimensional protein structure was modeled using Swiss-Model.

### Clinical interpretation

Variants were classified according to the standards and guidelines established by the American College of Medical Genetics and Genomics (ACMG) and the Association for Molecular Pathology (AMP) (15).

### Literature search strategy

To summarize the variant spectrum of the TNNT2 gene reported in Chinese patients with DCM, we conducted a comprehensive literature search up to November 30th 2025. Databases queried included PubMed, Web of Science, China National Knowledge Infrastructure (CNKI), and Wanfang Database. The search strategy utilized a combination of keywords and subject terms related to the gene (e.g., “TNNT2”, “troponin T2”), the disease (e.g., “dilated cardiomyopathy”, “DCM”), and the population (e.g., “Chinese”, “China”). All retrieved records were independently screened by researchers in our group based on titles and abstracts. Studies were included if they reported genetic testing results for TNNT2 in Chinese DCM patients (familial or sporadic). Conference abstracts and studies that did not explicitly describe the specific variant(s) were excluded. From the full texts of eligible studies, data on specific variants and the number of carrier cases were extracted. The pathogenicity of each variant was further assessed on the Franklin by Genoox platform (https://franklin.genoox.com) in accordance with the ACMG guidelines.

## Results

### Clinical data

**Figure 1:**
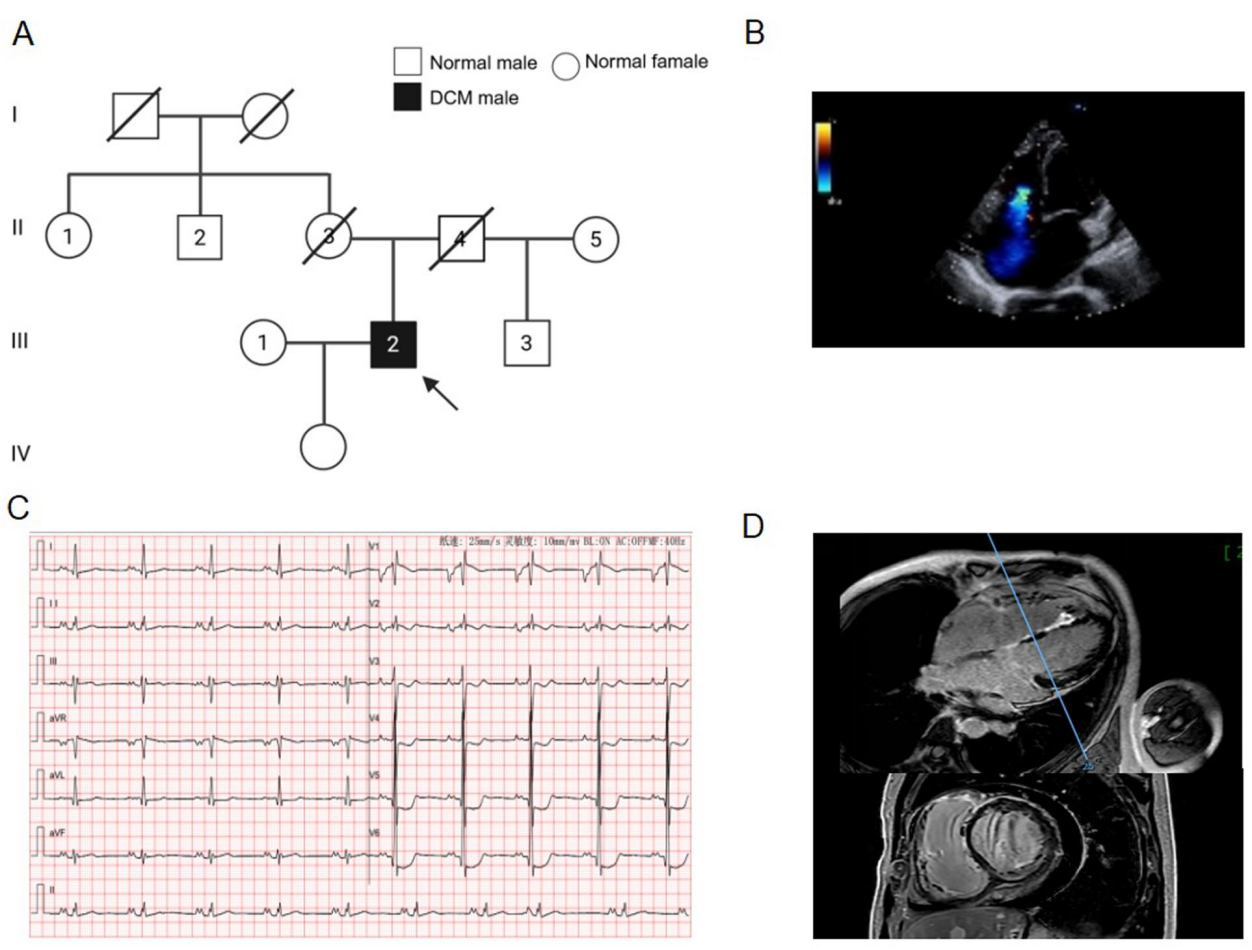
Clinical and pedigree data of the proband with DCM. **(A)** Roman numerals refer to the family generations. Squares represent male relatives; circles represent female relatives; the filled symbol indicates the DCM patient; slashes indicate deceased members; the arrow indicates the proband. **(B)** The echocardiographic image of the proband (four-chamber view). **(C)** Electrocardiogram image. (**D**) Images of cardiac magnetic resonance imaging.

The 41-year-old male proband presented with exertional dyspnea. He denied a history of hyperlipidemia, hypertension, or substance abuse. At presentation, blood pressure was 108/71 mmHg, and heart rate was 67 bpm. Genetic analysis of the proband's family revealed a history of sudden death in his mother at a young age (Figure 1A). Echocardiography revealed left atrial and ventricular enlargement, diffuse hypokinesis of the left ventricular walls, and left ventricular systolic dysfunction [left atrial diameter: 5.1 cm, left ventricular end-diastolic diameter: 5.9 cm, left ventricular ejection fraction (LVEF): 31%] (Figure 1B, Table 1). We observed elevated levels of high-sensitivity troponin I (29.3 ng/L) and BNP (2016.5 pg/mL). Viral serology and autoantibody screening (see Material and methods for the full panel) were negative. ECG showed sinus bradycardia, occasional atrial premature beats, delayed atrioventricular conduction, left atrial enlargement, left ventricular high voltage, incomplete right bundle branch block, and ST-T changes (Figure 1C). CMR revealed late gadolinium enhancement (LGE) in the basal to mid-septal and anterolateral segments of the left ventricle (Figure 1D).

**Table 1:** The Proband's echocardiographic data.

Clinical parameters	Measurements	Adult reference values
AAO (cm)	2.6	2.5–3.3
LA (cm)	5.1	2.7–3.5
LV (cm)	5.9	3.5–5.3
IVS (cm)	1.0	0.8–1.1
RA (cm)	4.5	3.2–4.5
RV (cm)	4.3	3.2–4.4
PA (cm)	2.1	2.4–2.8
LVFS (%)	15	>25
LVEF (%)	31	50–70

AAO, aorta ascendens; LA, left atrium; LV, left ventricle; IVS, interventricular septum; RA, right atrium; RV, right ventricle; PA, pulmonary artery; LVFS, left ventricular fractional shortening; and LVEF, left ventricular ejection fraction.

### Genetic screening and validation

**Figure 2:**
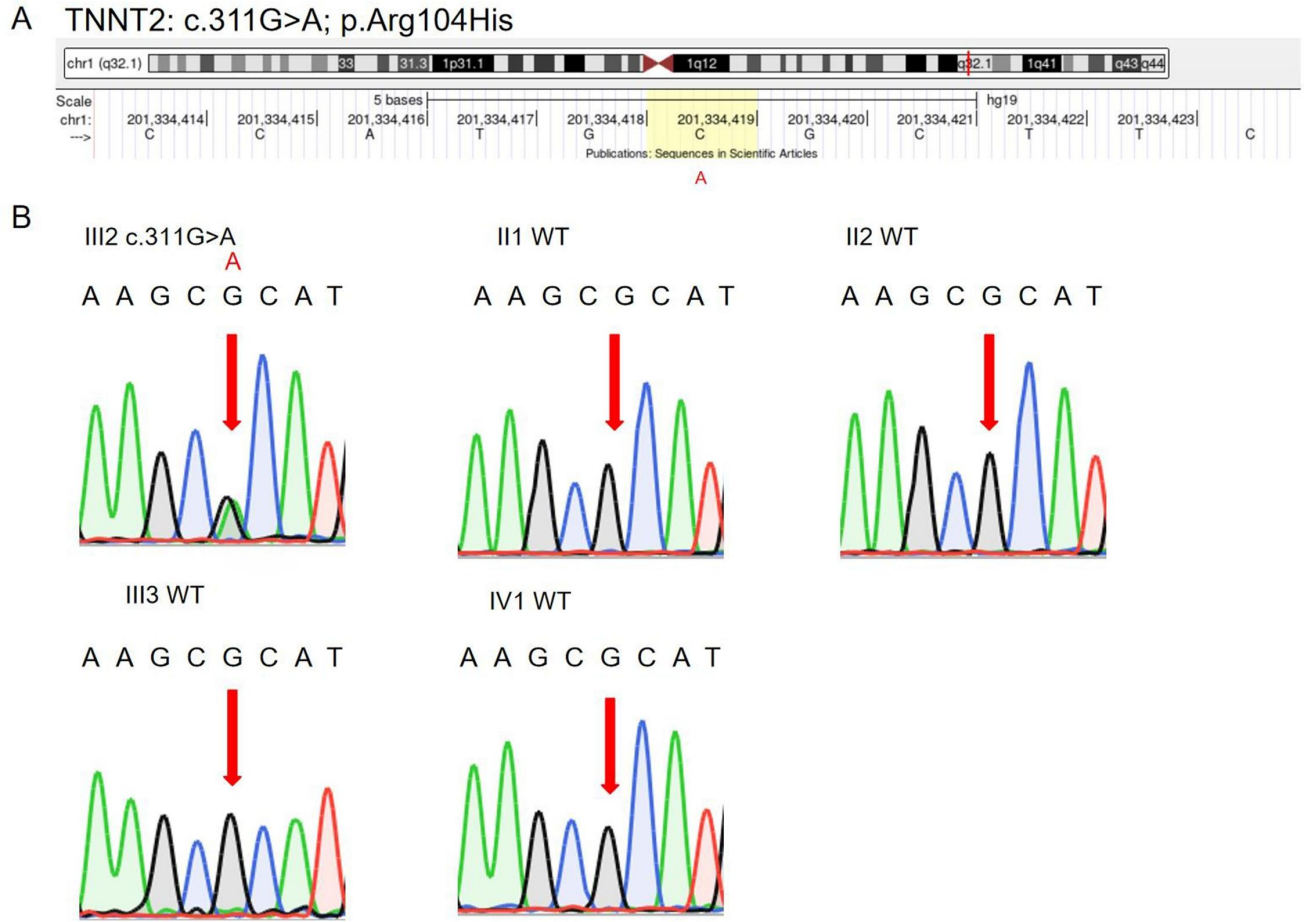
Localization and validation of TNNT2 c.311G > A (p.Arg104His) variant. **(A)** Schematic of the TNNT2 gene shows the location of the c.311G > A variant within exon 10 on chromosome 1. The specific nucleotide is highlighted with a yellow background, indicating the position leading to the p.Arg104His amino acid change. **(B)** Sanger sequence observed in the proband and relatives (II1, II2, III3, IV1). The arrow indicates the location of the variant.

**Figure 3:**
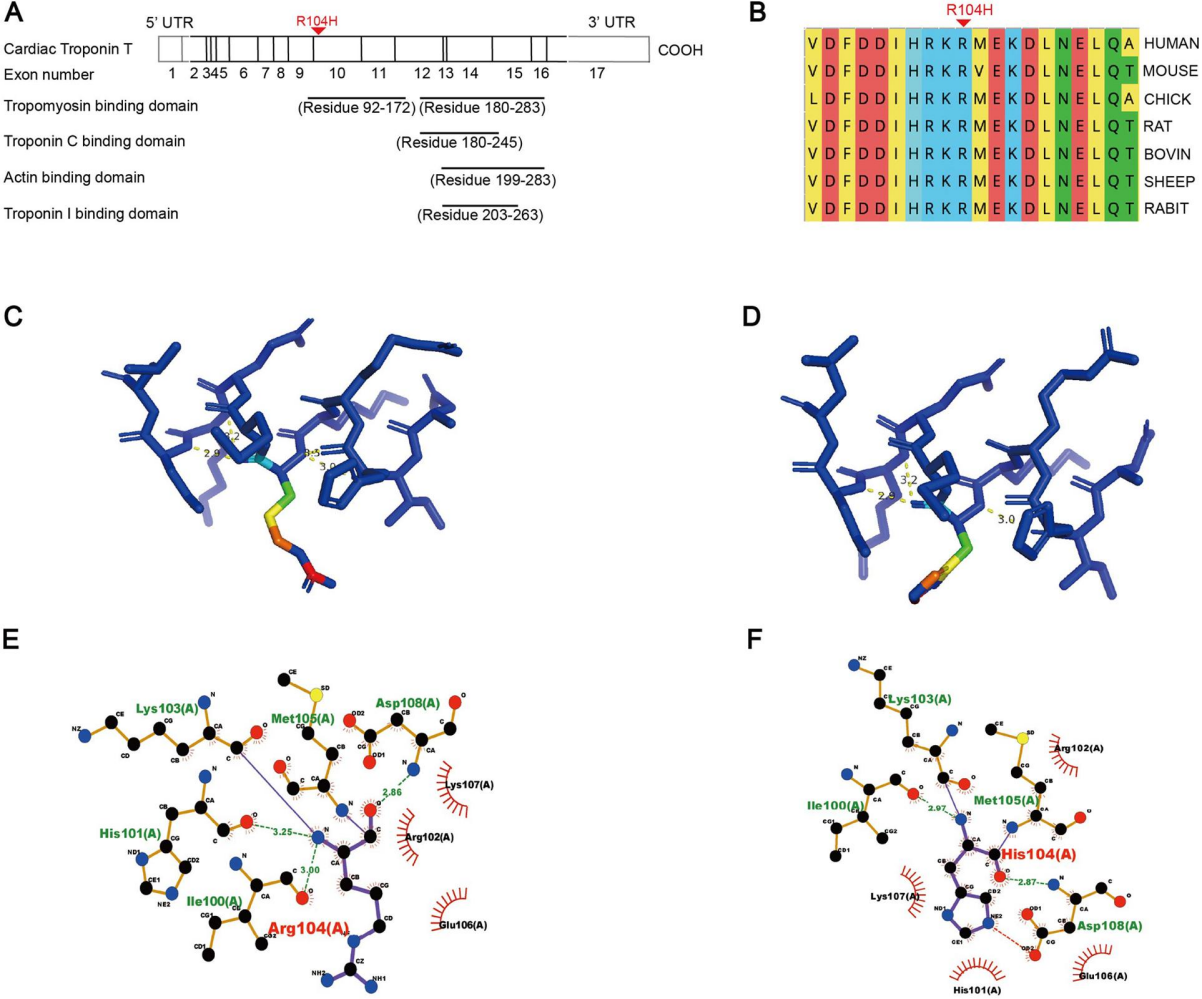
Conservation analysis, domain localization, and 3D structure analysis of the TNNT2 variant. **(A)** Schematic diagram of the TNNT2 protein domains. The red arrowhead indicates the location of the p.Arg104His variant within the tropomyosin-binding domain. **(B)** Multiple sequence alignment of the flanking region of the TNNT2 protein surrounding residue 104 across different species. The red arrow highlights the position of the p.Arg104His variant. **(C,D)** Three-dimensional structural modeling of the TNNT2 protein focusing on the local environment of residue 104 (**C**: wild-type, **D**: variant). The amino acid at position 104 is highlighted in red, and hydrogen bonds are represented by yellow dashed lines. (**E,F**) Two-dimensional visualization of molecular interactions between residue 104 and surrounding amino acids (**E**: wild-type, **F**: variant). Key residues are depicted using a ball-and-stick model, and hydrogen bonds are represented by green dashed lines. Residues involved in hydrophobic interactions are represented by arcs with radiating spokes.

WES and subsequent filtering identified a heterozygous missense variant in the TNNT2 gene (NM_001276345.2: c.311G > A, p.Arg104His) (Figure 2A). This variant was confirmed by Sanger sequencing in the proband. Sanger sequencing of available family members (daughter, half-brother, maternal uncle and maternal aunt) confirmed that none carried the variant, supporting its *de novo* origin (Figure 2B, Table 2). Protein analysis identified this variant within the tropomyosin-binding domain of TNNT2 (Figure 3A).

**Table 2:** Primers for PCR identification of TNNT2 c.311G > A variant.

Target	Forward primer	Reverse primer
TNNT2-Exon10	TTGATGTAAGCGGTGGCTGT	CCAGTAGGATGGGGAGGGAA

### *In silico* prediction

Comprehensive *in silico* analysis of the TNNT2: c.311G > A, p.Arg104His variant was performed. The variant is absent from major population databases (1000 Genomes, ESP6500, ExAC, gnomAD, gnomAD-EAS). Multiple sequence alignment demonstrates high evolutionary conservation at the variant site and its flanking regions (Figure 3B). Evolutionary conservation typically indicates critical structural roles, and variants in these regions frequently compromise structural stability and protein function. Tertiary structure modeling using Swiss-Model revealed that this variant reduces the hydrogen bonding between residue 104 and surrounding amino acids from four to three bonds, while increasing the hydrophobic interaction partners from three to four residues (Figures 3C–F). These *in silico* predictions suggest that the variant may affect protein conformation and function, though experimental validation is required. Based on the ACMG guidelines, the evidence from PS4, PM1_Supporting, PM2_Supporting, and PP3 supports its classification as “Likely Pathogenic”.

### Review

**Figure 4:**
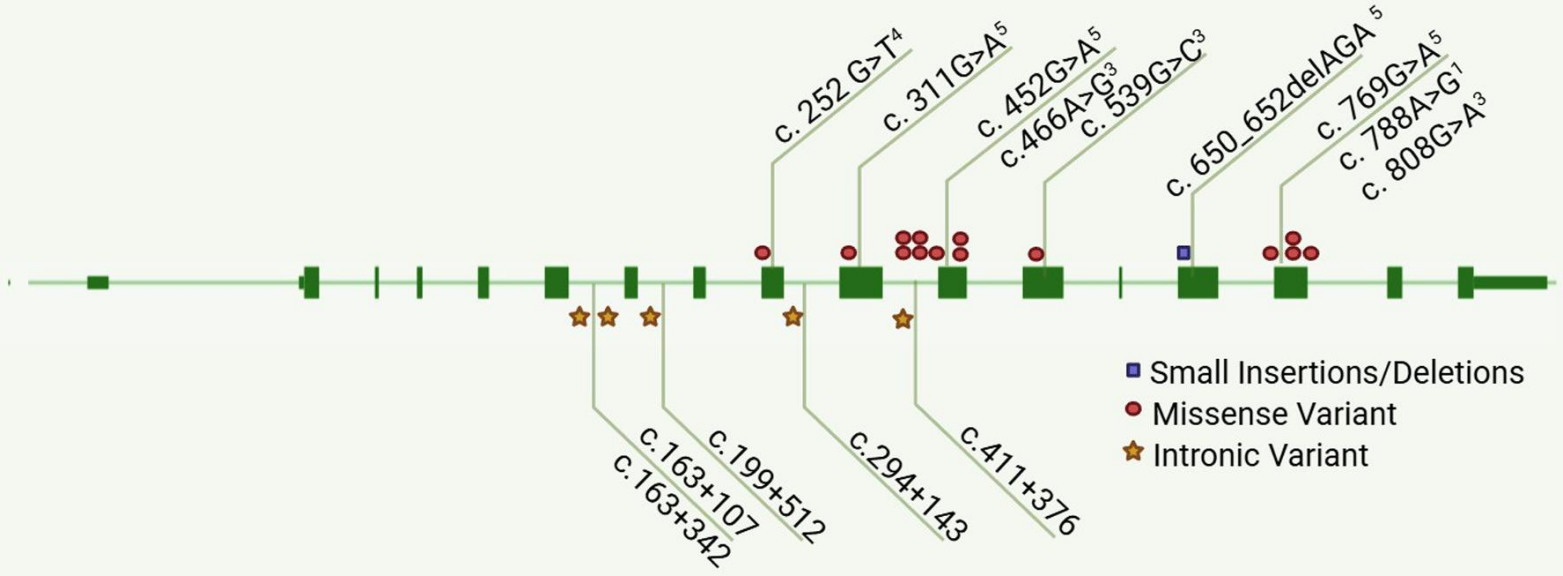
Spectrum of reported TNNT2 variants in Chinese patients with DCM. This summary figure presents the distribution characteristics of these TNNT2 variants using a gene structure visualization approach. Exons are depicted as green boxes, connected by lines representing introns, and the direction of the gene sequence is labeled from left to right (including the p.Arg104His variant identified in this study). Different types of variants are distinguished by symbols of specific colors and shapes. The number of symbols corresponds to the frequency of occurrence of the corresponding type of variant. The pathogenicity class of the variant is predicted on the Franklin website according to ACMG guidelines. 1, benign; 2, likely benign; 3, VUS (variants of uncertain significance); 4, likely pathogenic; 5, pathogenic. Intronic variants are not assigned a pathogenicity class here because the available evidence for most reported intronic variants is insufficient for a definitive ACMG classification; they are typically classified as VUS or lack published classification. The numbers next to intronic variant symbols denote their specific genomic positions relative to the nearest exon.

A comprehensive literature review identified 14 distinct TNNT2 variants among 20 reported Chinese DCM cases, including the present case. These comprised one small deletion, five intronic variants, and the remainder were missense variants. According to the pathogenicity classification on the Franklin platform, most of these variants were assessed as “Pathogenic” or “Likely Pathogenic.” Among them, only c.788A > G was classified as “Benign,” and c.539G > C, c.808G > A, and c.466A > G remained a “Variants of Uncertain Significance (VUS)” (Figure 4, Table 3).

**Table 3:** Summary of reported TNNT2 gene variants in Chinese patients with DCM.

Variant	Amino acid change	Variant type	Pathogenicity	Case count	Reference
c.252 G > T	p.Leu84Phe	Missense variant	Likely Pathogenic	1	PMID: 25110706
c.311 G > A	p.Arg104His	Missense variant	Pathogenic	1	Our Case
c.452 G > A	p.Arg151Gln	Missense variant	Pathogenic	5	PMID: 35284542; PMID: 39962380
c.466 A > G	p.Lys156Glu	Missense variant	VUS	2	PMID: 39962380
c.539 G > C	p.Gly180Ala	Missense variant	VUS	1	PMID: 26458567
c.650_652 delAGA	p.Lys217del	Small deletion	Pathogenic	1	PMID: 33941202
c.769 G > A	p.Glu257Lys	Missense variant	Pathogenic	1	PMID: 38054088
c.788 A > G	p.Lys260Arg	Missense variant	Benign	2	PMID: 35284542
c.808 G > A	p.Glu270Lys	Missense variant	VUS	1	PMID: 39962380
c.163 + 107C > T	Noncoding	Intronic variant	—	1	PMID: 25110706
c.163 + 342C > T	Noncoding	Intronic variant	—	1	PMID: 25110706
c.199 + 512A > C	Noncoding	Intronic variant	—	1	PMID: 25110706
c.294 + 143G > A	Noncoding	Intronic variant	—	1	PMID: 25110706
c.411 + 376A > G	Noncoding	Intronic variant	—	1	PMID: 25110706

VUS, variants of uncertain significance.

## Discussion

The advent of high-throughput next-generation sequencing (NGS) has accelerated the discovery of rare genetic variants. Using whole-exome sequencing and Sanger sequencing, we identified and characterized the TNNT2 c.311G > A (p.Arg104His) variant in a case of sporadic DCM. Although the proband had a family history of sudden death in the mother at a young age, the variant was absent in two of the mother's siblings, reducing the likelihood of maternal inheritance and suggesting a *de novo* variant event. This finding may hold significant clinical importance, providing not only a diagnostic basis for the disease but also clear guidance for genetic counseling within this family.

DCM is characterized by ventricular dilation and systolic dysfunction. Beyond cardiomegaly and impaired contraction, the diagnosis can be supported by specific arrhythmias, the presence of cardiac autoantibodies, endomyocardial biopsy results, or the identification of pathogenic gene variants (15). Our discovery underscores the value of genetic testing even in seemingly sporadic cases, as *de novo* variants can occur.

The TNNT2 c.311G > A (p.Arg104His) variant identified in our study has been previously annotated in ClinVar (VCV000043628) as likely pathogenic, although this classification is predominantly based on its established association with hypertrophic cardiomyopathy (HCM) (16, 17). Studies have shown that patients with HCM caused by TNNT2 variants often have a high risk of sudden death and relatively mild left ventricular hypertrophy (18, 19). Increased myofilament Ca^2+^ sensitivity is a key mechanism in HCM-associated TNNT2 variants, which impair the relaxation of cardiomyocyte and thus lead to a diastolic dysfunction (20–22). However, DCM-associated TNNT2 variants have been suggested to reduce myofilament Ca^2+^ sensitivity (22). A decrease in myofilament Ca^2+^ sensitivity impairs cardiomyocyte contraction, leading to compensatory left ventricular dilation to maintain cardiac output. The R141W variant associated with DCM, located in the tropomyosin-binding region of TNNT2, strengthens thin filament integrity by stabilizing the interaction between cTnT and tropomyosin, which leads to Ca^2+^ desensitization (23). In addition to the variant location, the dose of the mutant protein can also influence the alteration in calcium sensitivity. Experimental data from troponin exchange experiments in permeabilized cardiomyocytes demonstrate that the R278C variant enhances Ca^2+^ sensitivity at low and intermediate mutant protein dose, while reducing it at high dose (24). This dose-dependent effect suggests that the same variant could produce opposite functional consequences depending on the level of mutant protein incorporation, potentially leading to different disease phenotypes.

Research has revealed that TNNT2 gene variants directly regulate myofilament calcium sensitivity by altering the binding affinity between cTnT and tropomyosin. HCM-associated variants typically decrease the affinity of TnT for tropomyosin, whereas DCM-associated variants increase it. Notably, many of these functionally significant variants are located within the amino acid residues 92–144 (25, 26). Our identified variant, p.Arg104His, also lies within this critical region. Our *in silico* structural modeling suggests that this variant may disrupt local protein interactions (Figures 3C–F). It is therefore plausible to speculate that this variant may contribute to DCM pathogenesis by altering the TnT-tropomyosin binding affinity, thereby reducing myofilament calcium sensitivity. Our study paves the way for further mechanistic investigations to explore whether novel pathogenic pathways are involved.

Our review reveals a diverse spectrum of TNNT2 variants in the Chinese DCM population, including missense, intronic, and deletion variants (27). This distribution is consistent with that observed in other populations, with missense variants being the most frequent.

## Limitations

The conclusions of this study should be interpreted within the following limitations. For one thing, the absence of maternal DNA precludes definitive confirmation of a *de novo* origin, limiting the completeness of segregation evidence. For another, our evidence remains largely predictive. A definitive causal link between the p.Arg104His variant and DCM pathogenesis awaits establishment through functional validation in *in vitro* or *in vivo* models.

## Conclusion

This study provides a detailed clinical characterization of the TNNT2 c.311G > A (p.Arg104His) variant in a Chinese patient with sporadic DCM, contributing to the understanding of its phenotypic spectrum. These findings underscore the importance of genetic testing in sporadic cases and add to the evidence implicating TNNT2 in DCM pathogenesis. Furthermore, it provides a specific case to inform genotype-phenotype correlations for TNNT2-related cardiomyopathies.

## Data Availability

The original contributions presented in the study are included in the article. Further inquiries can be directed to the corresponding authors.
